# Automated segmentation and recognition of *C. elegans* whole-body cells

**DOI:** 10.1093/bioinformatics/btae324

**Published:** 2024-05-22

**Authors:** Yuanyuan Li, Chuxiao Lai, Meng Wang, Jun Wu, Yongbin Li, Hanchuan Peng, Lei Qu

**Affiliations:** Ministry of Education Key Laboratory of Intelligent Computation and Signal Processing, Information Materials and Intelligent Sensing Laboratory of Anhui Province, School of Electronics and Information Engineering, Anhui University, Hefei, Anhui 230039, China; Ministry of Education Key Laboratory of Intelligent Computation and Signal Processing, Information Materials and Intelligent Sensing Laboratory of Anhui Province, School of Electronics and Information Engineering, Anhui University, Hefei, Anhui 230039, China; Ministry of Education Key Laboratory of Intelligent Computation and Signal Processing, Information Materials and Intelligent Sensing Laboratory of Anhui Province, School of Electronics and Information Engineering, Anhui University, Hefei, Anhui 230039, China; Ministry of Education Key Laboratory of Intelligent Computation and Signal Processing, Information Materials and Intelligent Sensing Laboratory of Anhui Province, School of Electronics and Information Engineering, Anhui University, Hefei, Anhui 230039, China; College of Life Sciences, Capital Normal University, Beijing 100048, China; SEU-ALLEN Joint Center, Institute for Brain and Intelligence, Southeast University, Nanjing, Jiangsu 210096, China; Ministry of Education Key Laboratory of Intelligent Computation and Signal Processing, Information Materials and Intelligent Sensing Laboratory of Anhui Province, School of Electronics and Information Engineering, Anhui University, Hefei, Anhui 230039, China; SEU-ALLEN Joint Center, Institute for Brain and Intelligence, Southeast University, Nanjing, Jiangsu 210096, China; Institute of Artificial Intelligence, Hefei Comprehensive National Science Center, Hefei, Anhui 230039, China; Hefei National Laboratory, University of Science and Technology of China, Hefei, Anhui 230039, China

## Abstract

**Motivation:**

Accurate segmentation and recognition of *C.elegans* cells are critical for various biological studies, including gene expression, cell lineages, and cell fates analysis at single-cell level. However, the highly dense distribution, similar shapes, and inhomogeneous intensity profiles of whole-body cells in 3D fluorescence microscopy images make automatic cell segmentation and recognition a challenging task. Existing methods either rely on additional fiducial markers or only handle a subset of cells. Given the difficulty or expense associated with generating fiducial features in many experimental settings, a marker-free approach capable of reliably segmenting and recognizing *C.elegans* whole-body cells is highly desirable.

**Results:**

We report a new pipeline, called automated segmentation and recognition (ASR) of cells, and applied it to 3D fluorescent microscopy images of L1-stage *C.elegans* with 558 whole-body cells. A novel displacement vector field based deep learning model is proposed to address the problem of reliable segmentation of highly crowded cells with blurred boundary. We then realize the cell recognition by encoding and exploiting statistical priors on cell positions and structural similarities of neighboring cells. To the best of our knowledge, this is the first method successfully applied to the segmentation and recognition of *C.elegans* whole-body cells. The ASR-segmentation module achieves an *F*1-score of 0.8956 on a dataset of 116 *C.elegans* image stacks with 64 728 cells (accuracy 0.9880, AJI 0.7813). Based on the segmentation results, the ASR recognition module achieved an average accuracy of 0.8879. We also show ASR’s applicability to other cell types, e.g. platynereis and rat kidney cells.

**Availability and implementation:**

The code is available at https://github.com/reaneyli/ASR.

## 1 Introduction

The development of cell imaging and fluorescent labeling technologies has yielded unprecedented opportunities to study cellular development, fate, and function (Yemini *et al.* 2021). Two enabling computational techniques required to interpret these 3D microscopy images are automatic segmentation and recognition/identification of cells. Once cells are segmented and identified, their gene expression can be precisely quantified and compared across specimens and developmental stages, thereby facilitating genetic, developmental, and phenotypic assays at the single-cell level (Chaudhary *et al.* 2021, Nguyen et al. 2017, Yu *et al.* 2021).

**Figure 1.**
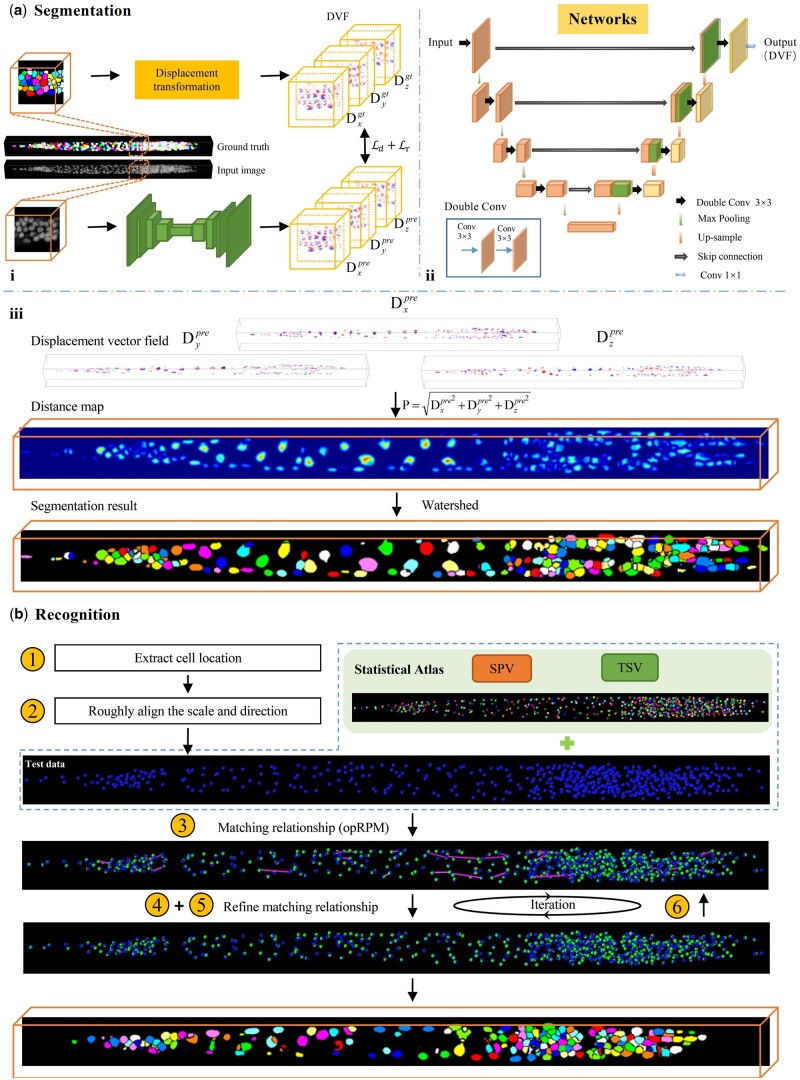
Overview of ASR pipeline. (a) Cell segmentation procedures. (i) Training process of segmentation network. (ii) Architecture of segmentation network. (iii) Convert the displacement vector field (DVF) generated by the network into a distance map, followed by the generation of the segmentation result using watershed (note that the color of each cell after watershed do not represent a specific identity). (b) Cell recognition procedures. The number in the yellow circle corresponding to the recognition step described in Section 2.2.2. The blue dots and green dots in the image represent predicted and ground truth cell positions, respectively. The purple line indicates the distance between the predicted position and the ground truth position. The orange box shows the final segmentation and recognition results.

*C.elegans* is an ideal organism for many developmental biological studies due to its invariant lineage and unique identities of cells (White *et al.* 1986, Yemini *et al.* 2021,). In particular, the newly hatched L1-stage *C.elegans* larva has stereotypically located 558 cells. However, the high-throughput segmentation and recognition of its cells remains a bottleneck. The challenge in whole-body cell segmentation lies in effectively separating cells that appear in dense clusters with inhomogeneous intensity profile and blurred boundaries (Fig. 1a and Supplementary Fig. S1). Despite the stereotyped location of cells in the L1-stage *C. elegans*, their dense distribution and variations in individual spatial arrangement are sufficient enough to make cell recognition another challenge.

Existing methods either can only handle a subset of sparsely labeled cells (Long *et al.* 2008, Qu *et al.* 2011, Kainmueller *et al.* 2014) or require additional fluorescent labeling in sample preparation (Yu et al. 2020, Yemini *et al.*, 2021). Early *C.elegans* cell identification methods mainly focused on the sparsely labeled non-neuronal cells, such as muscle cells (Long *et al.* 2008, 2009, Liu *et al.* 2009, Qu *et al.* 2011). With the development of all-in-one neuron identification strains, the requirement has shifted to neuron identification (Nejatbakhsh *et al.* 2020, Varol *et al.* 2020, Chaudhary *et al.* 2021). Differing from muscle cells, neurons in *C. elegans* are highly concentrated, especially in the head region, and exhibit a high degree of morphological similarity, making them extremely difficult to be separated and identified. Alternatively, some researchers resort to utilizing multicolor reporter strains and semi-automatic methods for neuronal identification (Yu *et al.* 2020, Emmons *et al.* 2021, Yemini *et al.* 2021). Due to the cost of building transgenic animals and limited fluorescent color channels, it is highly desirable to automate the whole-body cells segmentation and recognition without requiring additional fiducial patterns.

The accurate segmentation of cells is not only crucial for various biological analyses (e.g. gene expression quantification, cell development), but also forms the foundation for subsequent cell identification by providing essential features such as cell quantity, positions, and shape. General adaptive thresholding or watershed methods (Rahali *et al.* 2022) often lead to significant under-segmentation or over-segmentation. Additional cell grouping and splitting operations are generally required (Long *et al.* 2009). To better leverage the shape prior of cells, some methods modeled cells as ellipsoids and employed techniques such as elliptical fitting (Toyoshima *et al.* 2016), sliding band filter (Quelhas *et al.* 2010), Gaussian mixture models (Toyoshima *et al.* 2016), deconvolution (Yemini *et al.* 2021), etc., for cell segmentation. However, in scenario with densely packed cells, the results of these traditional methods still require additional manual optimization (Wen *et al.* 2021, Yemini *et al.* 2021, Li *et al.* 2023). Deep learning has thrived the field in recent years, bring substantial improvements in both precision and efficiency (Ahmad *et al.* 2022, Qadri *et al.* 2023). To better separate touched and overlapped cells, some methods emphasize cell boundaries by simultaneous regressing boundaries, background, and nuclei (Chen *et al.* 2016, Kumar *et al.* 2017, Cui *et al.* 2019), or by separately regressing cell and boundaries before fusion (Khoshdeli *et al*. 2018, Zhou *et al.* 2019). Since boundaries of *C.elegans* cells are typically blurry and dim in 3D fluorescence microscopy imaging, these methods cannot generalize well in our cases. Zhang *et al.*, (2022) elevated this issue by regressing the distance of each foreground voxel to its nearest background. Nevertheless, the relatively small size of *C.elegans* nucleus limits the effective learning of distance map. Despite recent tremendous successes of large-model in 2D cell segmentation, their application in 3D cases is still prohibited due to the limited size of training dataset of 3D cells (Greenwald *et al.* 2022).

Cell recognition aims to discern the unique identity of each individual cell, thus facilitating single cells targeting and improved throughput for genetic and phenotypic assays. The stereotyped spatial distribution of *C.elegans* cells (excluding ganglia cells) (White *et al.* 1986) is the most important prior for cell recognition. Long et al. proposed to build a digital atlas to encapsulate the spatial distribution of *C.elegans* cells, and realized the cell recognition by mapping atlas points to segmented cells (Long *et al.* 2009), or directly to image (Qu *et al.* 2011). The recognition performance was further improved by incorporating local position and shape priori (Kainmueller *et al.* 2014). However, due to inherent biological variability, spatial distribution of some cells in worms differs significantly from the positions of cells in atlas. Chaudhary *et al.* (2021) developed a data-driven consensus atlas that incorporates the priori about structural shapes between cells to further, improve the recognition performance. Nevertheless, the redundancy of prior information within the data-driven atlas and constraints on the number of cells limited its scalability.

In this article, we introduce an automated segmentation and recognition (ASR) pipeline. To the best of our knowledge, this is the first method successfully applied to the segmentation and recognition of 558 whole-body cells in 3D fluorescent microscopic images of *C.elegans* L1 larvae. We propose a novel displacement vector field (DVF)-based deep learning model to effectively segment densely packed cells with blurry and dim boundaries. By exploring and fully utilizing the statistical prior of cell positions and structural similarity, we present a statistical–structural matching-based method to achieve robust cell recognition in an iterative way. We experimentally evaluated the performance of our pipeline on datasets consisting of 116 3D image stacks, and demonstrated that our approach can generalizes well in producing reasonable automatic segmentation and recognition accuracy for 64 728 manually curated cells.

The rest of this article is organized as follows: Section 2 presents the framework of our proposed ASR, Section 3 shows the experimental results, and the conclusion is given in Section 4.

## 2 ASR algorithm

The ASR pipeline consists of two main modules: segmentation module and recognition module. The overall workflow of ASR is depicted in Fig. 1.

### 2.1 ASR_segmentation

#### 2.1.1 Displacement vector field

To better separate densely packed cells with blurred boundaries, unlike existing methods that directly regress the foreground label or distance map, we propose to regress the DVF for each cell using a deep neural network.

We define the DVF as the relative displacement of each voxel to its nearest background voxel. Let $\left(x_{i},y_{i},z_{i}\right)$ denote the coordinates of the *i*th voxel in the image and $\left(x_{i}^{\prime },y_{i}^{\prime },z_{i}^{\prime }\right)$ be the coordinate of its nearest background voxel. The displacement vector of the *i*th voxel is defined as:

(1) $${\vec{d}}_{i}=\{\begin{array}{cc} {}[0,0,0], & \text{if}\;i\text{th}\;\text{voxel}\;\in \Phi \\ {}[x_{i}-x_{i}^{\prime },y_{i}-y_{i}^{\prime },z_{i}-z_{i}^{\prime }], & \text{otherwise} \end{array}$$

where Φ denote background region. If a voxel belongs to Φ, we define its displacement vector as [0, 0, 0].

**Figure 2.**
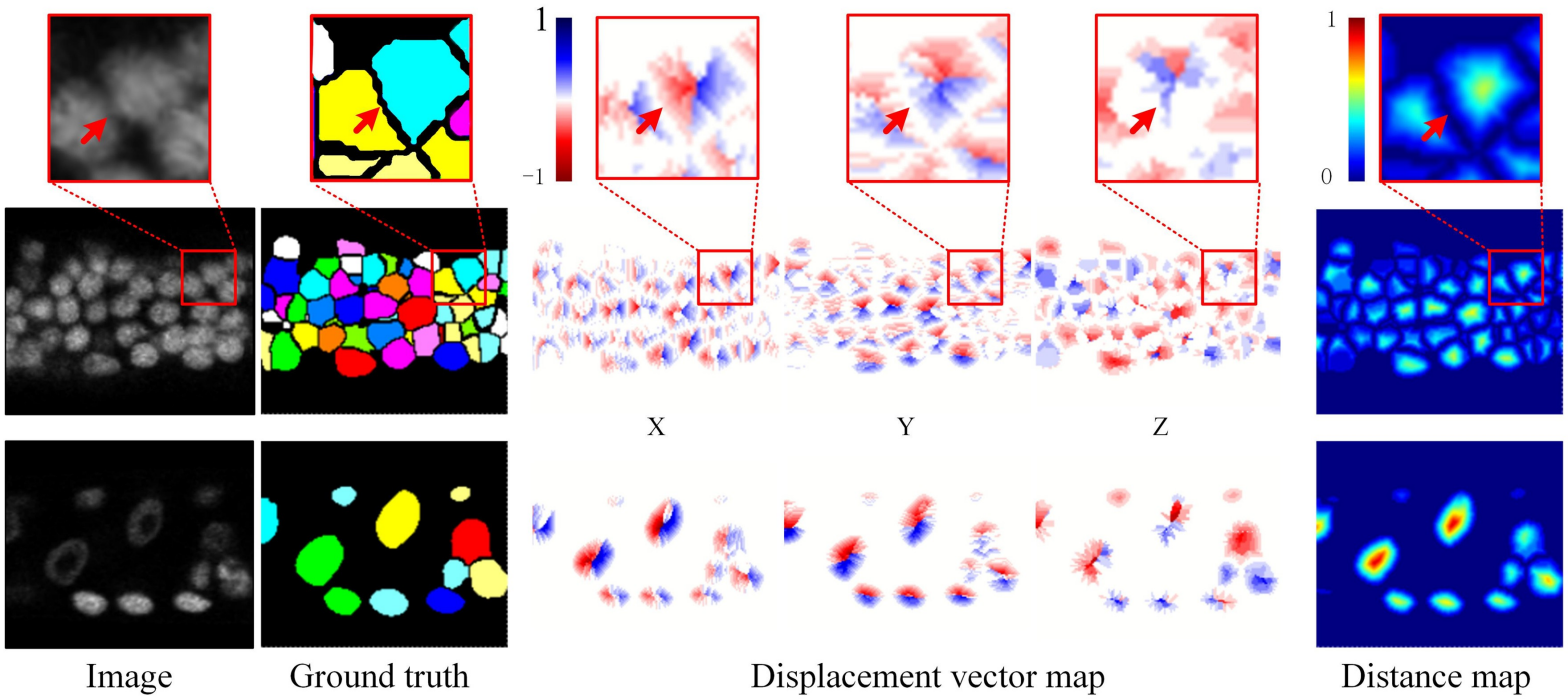
Displacement vector field. The first row shows the zoom-in view of the red boxes in the second row, with the red arrows pointing to the same coordinates. The second and third rows show the slice view (*XY* plane) of two different 3D image blocks in the original image, ground truth segmentation mask, displacement vector map, and distance map, respectively (Images are rescaled for better visualization.)

The DVF of input image stack comprises three channels, $D_{x},\;D_{y}$, and $D_{z}$, which correspond to the displacement in *x*, *y*, and *z* directions, respectively. Each channel has the same size as the input image stack (Fig. 1ai). As shown in Fig. 2, the displacement vectors at the boundary of adjacent cells exhibit completely different directions. The red arrows in Fig. 2 point to the same coordinates in the original image, image segmentation mask, displacement vector map, and distant map. The transition from blue to red in the three channels of the displacement vector map highlights the shift in the direction of the displacement vector from positive to negative values across the boundaries of adjacent cells. This will encourage the network to learn more discriminative features, thus facilitating the effective segmentation of densely packed cells. In addition, the DVF can be synthesized into the distance map **P** by calculating the *L*2 norm. This allows the DVF to not only preserve the advantages of the distance map, such as attention to the cell nucleus and cell shape, but also facilitate the generation of segmentation results through post-processing.

#### 2.1.2 Network and loss function

We employed a slightly modified 3D U-Net (Çiçek *et al.* 2016) to regress the DVF. Considering the small size of whole-body cells and the large volume of 3D fluorescence microscopy images of *C.elegans*, we chose to partition the image stack into smaller cubes before feeding to the network. We selected a cube size of 80×128×128 (depth–height–width) to strike a balance between hardware limitations and regression accuracy. The output DVF of each cubes will be assembled in the same order to generate the complete DVF. The detailed architecture of the network is shown in Fig. 1aii, which contains an encoder–decoder structure. Both the encoder and decoder stages have five layers with 32, 64, 128, 256, and 512 channels, respectively.

The loss function *L* of our network contains two components, a displacement vector loss *L_d_* and a directional loss *L_r_*:

(2) $$L=\omega_{1}L_{d}+\omega_{2}L_{r}$$

where *L_d_* is the mean squared error between the predicted and the ground truth DVF, and *L_r_* represent mean cosine similarity loss. *ω*_1_ and *ω*_2_ are two weighting parameters that determined experimentally. Given the input image $I\in R^{n\times m\times k}$, and the final output of the network is mapped to $R^{n\times m\times k\times 3}$. *L_d_* and *L_r_* can be written as:

(3) $$L_{d}=\frac{1}{3\times N}\sum_{i=1}^{N}∥{\vec{d}}_{i}^{\mathrm{pre}}-{\vec{d}}_{i}^{gt}{∥}^{2}$$

(4) $$L_{r}=1-\frac{1}{N}\sum_{i=1}^{N}\frac{{\vec{d}}_{i}^{\mathrm{pre}}\cdot {\vec{d}}_{i}^{gt}}{∥{\vec{d}}_{i}^{\mathrm{pre}}∥\cdot ∥{\vec{d}}_{i}^{gt}∥}$$

where N=n×m×k, and the superscripts *pre* and *gt* denote the predicted and ground truth displacement vectors, respectively.

With predicted DVF, as shown in Fig. 1aiii, we generate cell segmentation results by first transforming the DVF into the distance map ***P***, and then thresholding the distance map to generate the seeds for watershed segmentation (Naylor *et al.* 2018).

### 2.2 ASR_recognition

The key to *C.elegans* cell recognition lies in fully exploring and utilizing the stereotyped spatial distribution of cells, while effectively handling individual variations and cell localization errors introduced during segmentation. To address this challenge, we introduced a statistical–structural matching-based cell recognition method. Firstly, an informative whole-body cell statistical atlas of *C.elegans* is built to encapsulate statistic priors of cells distribution and the local topological relationships of adjacent cells. Subsequently, these statistical priors are effectively integrated into an iterative pipeline to achieve robust cell recognition.

#### 2.2.1 Statistical atlas

**Figure 3.**
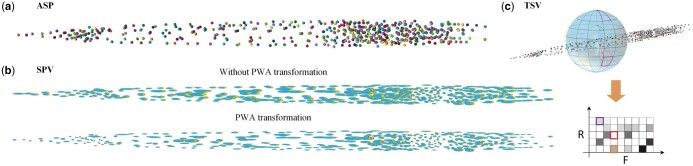
Statistical atlas of *C.elegans* whole-body cells. (a) ASP: the average spatial position of each cell. (b) SPV: the variation of the position of each cell is visualized as an ellipse. (c) TSV: the local topological feature of each cell.

The statistical atlas of *C.elegans* we designed contains three parts (Fig. 3): average spatial positions (ASP), spatial position variation (SPV), and topological structure variation (TSV). ASP encodes the overall cell spatial distribution by calculating and recording the average spatial position of each whole-body cell across samples. While SPV models the spatial variations of each cell by computing the standard deviation of cell’s location along the *x*, *y*, and *z* directions, respectively. As a complement to ASP, TSV uses shape context (Frome *et al.* 2004) to capture the local topological relationships of neighboring cells. We generate this statistical atlas based on 464 manually annotated L1 larval image stacks. Detailed methods for generating this statistical atlas are outlined as follows:

**ASP:** The location of each cell in ASP is represented by a 3D point that statistically has the smallest displacement from the corresponding cell in all globally aligned samples in the training dataset. We generate the ASP using the following four steps. Firstly, an arbitrary sample in the training dataset is selected as the initial ASP, and the remaining samples are globally aligned to the ASP using 3D affine transformation. The centroids of corresponding cells in all aligned samples are then averaged to produce a new ASP. Secondly, the average deformation field of all samples is computed, inverted, and applied to deform the ASP generated in the first step. This step is designed to compensate the bias of ASP toward to the initially selected target sample in atlas building. Thirdly, we go back to step one, replace the initial ASP with the deformed ASP generated in step two, and iterate until converge. Lastly, we globally map the cells in each sample to the ASP using piecewise affine (PWA) transformation (Nakada *et al.* 2005). The mapped results are averaged to generate the final ASP *(*as shown in Fig. 3a). Considering the image stacks of *C.elegans* are noticeable longer in the axial direction, the employment of PWA will generate more reasonable results in both ASP and SPV compared to the global 3D affine transformation (Fig. 3a and b).

**SPV:** Based on the PWA mapped cell positions of all samples obtained in the ASP construction step, we calculate the standard deviation of each cell’s position along the *x*, *y*, and *z* directions to generate the SPV. Compared to the global 3D affine transformation, the utilization of PWA transformation considerable reduces the standard deviation of cells (Fig. 3b), thus offering more precise guidance for subsequent cell recognition.

**TSV:** We generate TSV feature for each cell in the atlas using the following two steps. First, the shape context feature (Frome *et al.* 2004) of each cell in the ASP and PWA aligned samples are calculated. Secondly, for each cell in the ASP, its shape context similarities to the corresponding cells in PWA aligned samples are calculated and averaged. The resulting averaged shape context similarity is then assigned as the TSV feature for that cell in the atlas. As shown in Fig. 3c, we calculate the shape context feature by first constructing a logarithmic polar coordinate system around the current cell centroid. This system consists of *R* concentric spheres in the radial direction, and all concentric spheres are divided into *F* sectors. Subsequently, a histogram is generated by counting the number of points in each spherical sector. We take this histogram as the shape context feature, and employ chi-square distance to quantify the similarity between shape contexts of corresponding cells.

#### 2.2.2 The pipeline of recognition

We realize the cell recognition by establishing one-to-one matches between segmented cells and ASP pointset while leveraging priors embedded in the atlas. For each newly straightened and segmented larva image stack, the process of cell recognition is as follows (see in Fig. 1b):

1. *Extract cell location*. Given segmented image stack, we extract the cell location by calculating the centroid of each segmented cell. We take the cell centroids as the subject pointset and the ASP as the target pointset.
2. *Roughly align the scale and direction of subject and target pointset*. Three principal axes of target and subject pointset were extracted using principal component analysis (PCA) and rigidly aligned.
3. *Establish the initial matching between subject and target pointsets*. We employ the deterministic annealing framework of robust point matching (RPM) (Chui and Rangarajan 2003) to establish the initial matching. In the original RPM, the matching matrix is constructed solely based on the spatial location similarity of the pointset. To fully leverage topological priors, in our modified RPM, we define the density function for calculating the similarity of the point-pair as:
  (5) $$f(s_{n}|v_{m})=\frac{1}{\alpha \beta }\text{exp}\;\left[-\left(\frac{d(s_{n},v_{m})}{\alpha }+\frac{g(s_{n},v_{m})}{\beta }\right)\right]$$
  where *v_m_* is the *m*th point in target points, and *s_n_* is the *n*th point in subject points. The function d(.) denotes the point-to-point Euclidean distance, and g(.) denotes the chi-square distance of the shape context features of *s_n_* and *v_m_*. The *α* and *β* control the contribution weights of spatial location and shape context features in similarity measures, respectively. In the annealing framework, *α* serves as a decay parameter, while *β* functions as an incremental parameter. This implies that spatial location plays a crucial role at the initial stage, whereas shape context features achieve fine-tuning during the later stage. The initial values of *α* and *β* are set through ablation experiments.
4. *Affine align the subject and target pointset*. Based on the initial matching results obtained in step (3), we sequentially calculate the 3D affine transformation and PWA transformation between the two pointsets, and then use them to map the subject points so that they are as close as possible to the corresponding target points.
5. *Refine the matching relationship*. Based on the affine-aligned results obtained in step (4), we further refine the matching relationship between subject and target pointsets by employing a bipartite matching-based method capable of fully leveraging the SPV and TSV priors. We use anisotropic Gaussian to model the matching probability density functions of each point-pair between subject and target. For each point-pair, two probability density functions, *f^spv^* and *f^tsv^*, are constructed to embed the SPV and TSV priors separately:
  (6) $$f^{\mathrm{spv}}(s_{n}|v_{m})=\text{exp}\;\left[-\frac{1}{2\omega^{2}}d(s_{n},v_{m},\sigma_{m})\right]$$
  (7) $$d(s_{n},v_{m},\sigma_{m})={\left\|\frac{s_{n}-v_{m}}{\sigma_{m}}\right\|}^{2}$$
  (8) $$f^{\mathrm{tsv}}(s_{n}|v_{m})=\text{exp}\;\left[-\frac{1}{2\omega^{2}}\frac{g{\left(s_{n},v_{m}\right)}^{2}}{{\left(\gamma_{m}\right)}^{2}}\right]$$
  where *σ*_*m*_ and *γ*_*m*_ denote the SPV and TSV prior information for the *m*th points in the statistical atlas, respectively. *ω* is a constant parameter. The matching matrices $F_{N\times M}^{\mathrm{spv}}$ and $F_{N\times M}^{\mathrm{tsv}}$ are generated by calculating the similarity between each subject point and the target pointset based on *f^spv^* and *f^tsv^*, respectively. The new matching relationship was obtained by solving a bipartite graph matching problem using Hungarian algorithm on the matching matrices. To minimize the mismatch, we apply the Hungarian algorithm on $F_{N\times M}^{\mathrm{spv}}$ and $F_{N\times M}^{\mathrm{tsv}}$ separately to obtain two sets of matching relationships between subject and target pointset. Their consensus matches are selected to replace the matching relations required in step (4).
6. Return to step (4) and iteratively refine the matching relationships until converge. The ultimate matching relationship between the two pointsets will be determined by applying the Hungarian Algorithm on $F_{N\times M}^{\mathrm{spv}}+F_{N\times M}^{\mathrm{tsv}}$. Given that the atlas includes the identity of each cell, the recognition of each cell in the new larva can be accomplished through the established matching relationships.

## 3 Experiments

### 3.1 Datasets and training configuration

Our *C.elegans* dataset comprises 580 image stacks of newly hatched L1 larvae. A detailed description of the dataset is provided in the Supplementary material. Each image stack was computational straightened, and the nuclei mask of each larvae was obtained through the pipeline provided by Long *et al.* (2009) and manually corrected. The size of each image was relatively consistent, with an average size of 128×144×1400. We randomly selected 464 images for training and the remaining 116 images for testing. Each image was partitioned into approximately 10 000 cubes before being fed into the network.

We conduct the ASR_segmentation experiments on machine equipped with an Intel i7-6700 CPU and an NVIDIA GeForce GTX TITAN GPU (12GB Memory). The ASR_segmentation was implemented using PyTorch (Ketkar and Moolayil 2021). The parameter settings used in experiments are described in the Supplementary material.

### 3.2 Evaluation metrics

We evaluate the performance of cell segmentation following the same metrics using by Cui *et al.* (2019) and Naylor *et al.* (2018), including accuracy, precision, recall, F1, AJI. Considering that the segmentation errors of small cells have little impact on above mentioned metrics. For a fairer comparison, in addition to the above metrics, we introduce a new metric called Instance-Intersection-over-Union (IIoU). IIoU first calculates the Intersection-over-Union (IoU) of each cell, and then calculates the mean of IoU of all cells in the images to ensure that the segmentation accuracy of cells of different sizes is equally considered. IIoU can be written as:

(9) $$\text{IIoU}=\frac{1}{M}\sum_{j=1}^{M}\frac{\left|G_{j}\cap P_{j}\right|}{\left|G_{j}\cup P_{j}\right|}$$

where *M* denotes the number of cells, $G_{j}$ denotes the GT segmentation mask of the *j*th cell, and $P_{j}$ denotes its predicted mask.

We use average precision (AP) (Chen *et al.* 2018, Schwendy *et al.* 2020) as a metric to evaluate the cell recognition performance. We report and compare the AP, AP@0.5, and AP@0.75 of different methods. AP@0.5 (or AP@0.75) means using an IoU threshold 0.5 (or 0.75) to identify whether a predicted mask is positive in the evaluation. AP indicates the average of results that obtained with ten evaluation thresholds rising from AP@0.5 to AP@0.95 in 0.05 steps.

### 3.3 Results and analysis

#### 3.3.1 ASR_segmentation performance evaluation

We evaluated the segmentation performance by comparing our method with seven segmentation algorithms: IFT-Watershed (Lotufo *et al.* 2002), Unet (Caicedo *et al.* 2019), Dist (Naylor *et al.* 2018), Cellpose3D (Eschweiler *et al.* 2022), 3DCellSeg (Wang *et al.* 2022), EMBEDSEG (Lalit *et al.* 2022), and SRS (Qu *et al.* 2011). To conduct a fair comparison, all methods (except SRS and IFT-WATERSHED) were trained and tested on partitioned datasets. SRS requires a complete image as input due to its atlas-to-image mapping mechanism and IFT-WATERSHED is not limited by GPU memory. The optimal weight parameters *ω*_1_ and *ω*_2_ in Equation (2) were determined experimentally (Supplementary Table S3), and we fixed *ω*_1_ = 7 and *ω*_2_ = 1 in all subsequent experiments. The learning curve of the ASR_segmentation model is shown in Supplementary Fig. S2.

**Figure 4.**
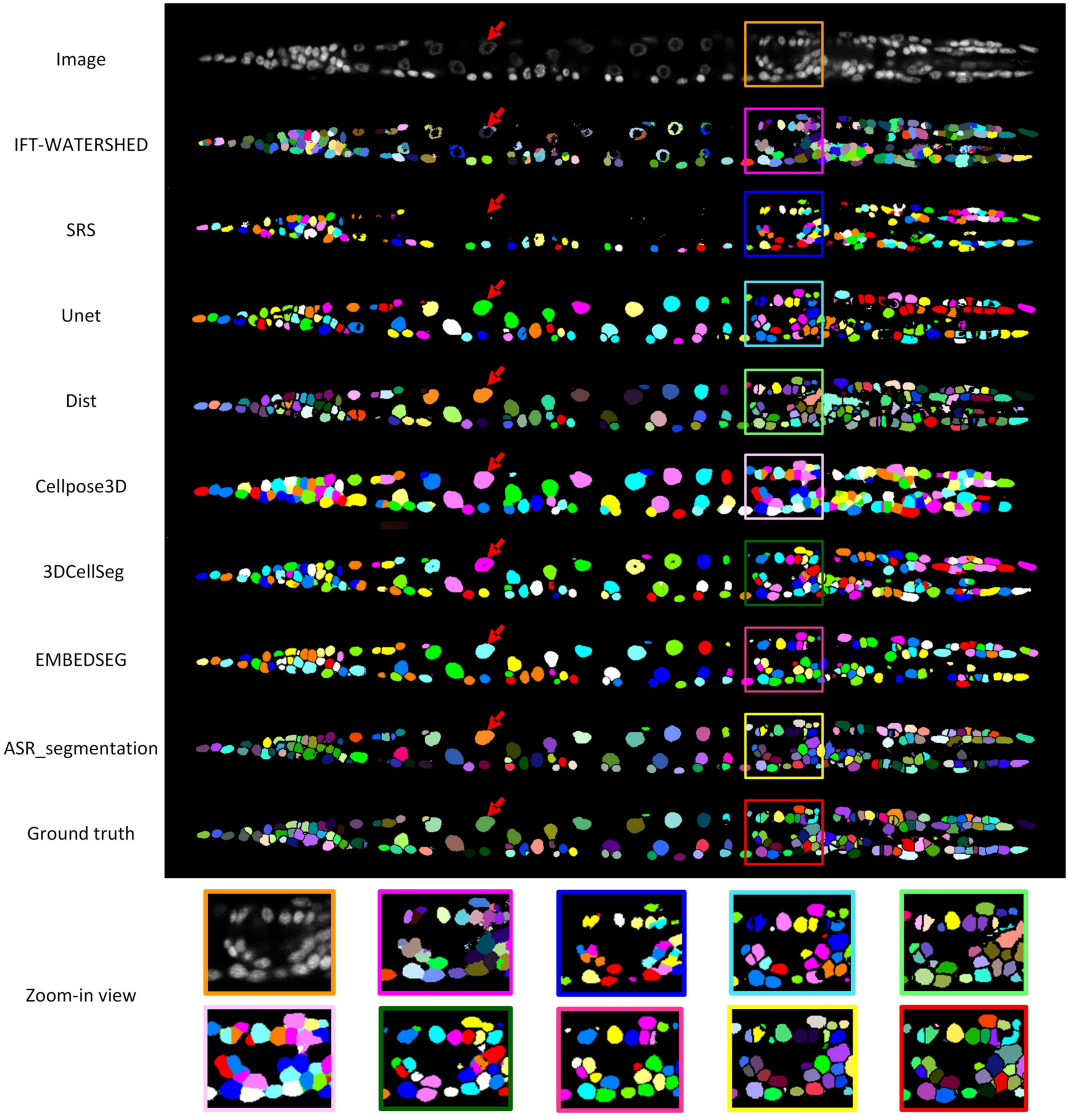
Comparison of the segmentation results of different methods. Each subfigure shows a slice view (*XY* plane) of the corresponding 3D image. Segmented cells are visualized using different colors. The red arrows pointing to the same coordinate.

The segmentation performance of different methods is quantified in Table 1. We can observe that the deep learning-based methods (Unet, Dist, Cellpose3D, 3DCellSeg, EMBEDSEG, ASR_segmentation) exhibit consistently superior performance compared to conventional methods (IFT-WATERSHED and SRS). Although SRS shows excellent performance in the case of sparse cell distribution, it is not well adapted to the segmentation of densely distributed whole-body cells. Our ASR-segmentation achieves the best results in accuracy, F1, AJI, and IIoU. Although Dist and Cellpose3D achieve the best results in terms of precision and recall, their low F1 scores indicate that they suffer from the missed and incorrect detections, respectively. These results confirmed that ASR_segmentation can effectively handle the segmentation of densely distributed cells. Similar conclusions can be drawn from Fig. 4, where segmentation results of different methods are visualized.

**Table 1.** Segmentation performance of different comparison methods.

Method	Accuracy	Precision	Recall	*F*1	AJI	IIoU
IFT-WATERSHED	0.9610	0.6181	0.5956	0.6067	0.1676	0.1887
SRS	0.9635	0.8878	0.4381	0.5824	0.3405	0.3934
Unet	0.9820	0.9405	0.7521	0.8373	0.6473	0.6607
Dist	0.9825	**0.9436**	0.7664	0.8614	0.7065	0.7101
Cellpose3D	0.9663	0.6503	**0.9047**	0.7560	0.5302	0.5413
3DCellSeg	0.9875	0.9007	0.8830	0.8911	0.7027	0.7047
EMBEDSEG	0.9872	0.9127	0.8632	0.8867	0.7625	0.7632
ASR_segmentation without partition	0.9817	0.8784	0.7927	0.8329	0.6326	0.6383
**ASR_segmentation**	**0.9880**	0.9401	0.8860	**0.8956**	**0.7813**	**0.7811**

Note: The best values in each metric are highlighted in bold.

Additionally, we confirmed that partition strategy significantly enhance segmentation performance, with IIoU improving from 0.6383 to 0.7811. In Supplementary Tables 4 and 5, we demonstrated that ASR_segmentation can also be successfully applied to the segmentation of other cell types, such as platynereis and rat kidney cells.

#### 3.3.2 ASR_recognition performance evaluation

We use the testing dataset (as described in Section 3.1) that contains 116 image stacks to evaluate the recognition accuracy of ASR. There are totally 64 728 manually annotated whole-body cells in the dataset. By concatenating the ASR_segmentation and ASR_recognition modules, we achieved a recognition accuracy (AP@0.5) of 0.8879 (as shown in Table 2). Compared with SRS, ASR shows a recognition accuracy improvement of about 0.3 measured in AP@0.5.

**Table 2.** ASR_recognition performance evaluation and the contribution of different statistical priors.

Method	AP	AP@0.5	AP@0.75
Cellpose3D + ASR_recognition	0.6472	0.6726	0.6541
EMBEDSEG + ASR_recognition	0.8460	0.8530	0.8475
Unet + ASR_recognition	0.6513	0.6621	0.6534
Dist + ASR_recognition	0.8330	0.8435	0.8342
3DCellSeg + ASR_recognition	0.6510	0.6676	0.6523
SRS	0.4827	0.5935	0.4724
ASR (ASP-RPM)	0.7894	0.7982	0.7886
ASR (ASP-opRPM)	0.8332	0.8424	0.8325
ASR (ASP-opRPM_SPV)	0.8702	0.8800	0.8695
**ASR (ASP-opRPM_SPV_TSV)**	**0.8781**	**0.8879**	**0.8773**

Note: The best values in each metric are highlighted in bold.

Unfortunately, other *C. elegans* cell recognition methods (Long *et al.* 2008, Qu *et al.* 2011, Kainmueller *et al.* 2014, Yu *et al.* 2020, Yemini *et al.* 2021) cannot be adapted to the recognition of *C. elegans* whole-body cells, either due to their lack of the whole-body cells priors or the requirement for recognition on specific fluorescently labeled data. In addition, we also compared the performance of ASR_recognition by concatenating with different cell segmentation algorithms including Cellpose3D, EMBEDSEG, Unet, Dist, 3DCellSeg. The comparison results shown in Table 2 further demonstrate the superior performance of ASR_segmentation from another aspect.

To verify the contribution of different statistical priors and topological structure information we used in ASR. We conducted a comprehensive ablation study and the results are shown in Table 2. We employ ASR (ASP-RPM) and ASR (ASP-opRPM) to denote recognition based on the original RPM and optimized RPM (Equation (5)), respectively, utilizing solely the ASP prior. Furthermore, ASR (ASP-opRPM_SPV) indicates the joint use of ASP and SPV priors, while ASR (ASP-opRPM_SPV_TSV) denotes all priors were used. We can notice that the introduction of SPV brings about an accuracy improvement of 0.038 (AP@0.5) and 0.037 (AP@0.75). When combining the two statistical priors, SPV and TSV, the recognition accuracy (AP) reaches 0.8781.

## 4 Conclusion

In this study, we present ASR, a high-throughput pipeline for automatic segmentation and recognition of 3D whole-body cells in L1 larvae. Distinguishing itself from existing algorithms that either identify only a subset of cells or require fluorescent labeling, ASR demonstrates the capability to recognize whole-body cells in unlabeled fluorescence microscopy images. We show that the introduction of the DVF effectively promotes the separation of densely distributed cells with blurred boundaries. Furthermore, through iterative optimization of the cell-to-atlas matching and mapping, we not only harness various priors embedded in the atlas but also achieve more robust cell recognition. However, the task independence inherent in ASR can occasionally be susceptible to segmentation errors, which may impact recognition accuracy. With the advancements in deep learning, deep learning-based *C.elegans* cell recognition is becoming increasingly feasible. We anticipate that depth learning-based joint segmentation and recognition of *C. elegans* cells will be realized in the future, further enhancing both accuracy and efficiency. Although our primary focus in this article is on 3D images, ASR’s adaptability to 2D images broadens its applicability, facilitating its use across various domains, including cell tracking.

## Supplementary Material

btae324_Supplementary_Data
