# Supplementary material for: Automated segmentation and recognition of C. elegans whole-body cells
Source: Bioinformatics. 2024 May 22;40(5):btae324. doi: 10.1093/bioinformatics/btae324 (PMC11139520; doi:10.1093/bioinformatics/btae324)
Supplement: btae324_Supplementary_Data [file btae324_supplementary_data.pdf]

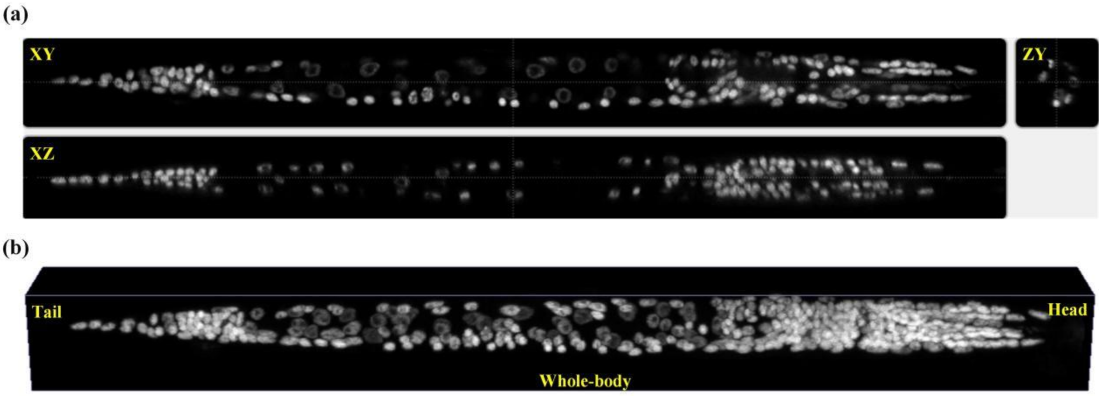

Supplementary Figure 1：L1-stage *C. elegans* image in the dataset. (a) Three-dimensional plane view of *C.elegent* image: XZ, ZY, XY. (b) Maximum density projection of *C. elegent* images.

C. elegans dataset introduction

All *C. elegans* strains were raised on Nematode Growth Media plates and fed OP50 *E. coli* as described (Girardet et al., 2007) at 20 °C in incubators. Worms hatched within three hours were collected as early stage L1 larvae. Worm fixation and DAPI-staining used a protocol modified from previous publication (Liu et al. 2009). Briefly, worms were washed by M9, spun down, and then quickly re-suspended by 4% PFA in Modified Ruvkun's Witches Brew (MRWB) and frozen in liquid nitrogen overnight. Worms were thawed at 4 °C, rotating at least 2 h, washed by Tris-Triton Buffer (TTB) with 100 mM DTT for 5 min, and then stained by DAPI or Hoechst at 1 µg/mL for 3 h. Stained worms were washed by TTB for 5 times and mounted in 60 % glycerin for microscopy. 3D image stacks of L1 larvae were obtained using a Zeiss confocal microscope with a ×63 oil objective (NA = 1.4). X-Y and Z dimension sampling was set at 0.116 µm and 0.122 µm per pixel, respectively.

Experimental details

In the ASR experiments, the settings for relevant parameters are available in Supplementary Table 1. The parameters $\boldsymbol{\alpha}$ and $\boldsymbol{\beta}$ in the density function formula for recognition (Eq. 6 in the main text) were weight parameters. To achieve optimal recognition performance, we conducted experiments as outlined in Supplementary Table 2. Based on Supplementary Table 2, we set $\boldsymbol{\alpha}$ and $\boldsymbol{\beta}$ to 0.05 and 0.06, respectively.

Our proposed segmentation method ASR_segmentaion excels in handling dense and ambiguously outlined C. elegans cells, but is applicable to other cells as well. In order to verify the performance of our proposed method on different types of cell data, we performed experiments on Platynereis-Nuclei-CBG (Chen et al. 2022) and BABB-cleared rat kidney (Lalit et al. 2021) datasets, respectively.

Supplementary Table 1. Experimental parameter setting

Parameter	Setting
Training epochs	90
optimizer	SGD
initial learning rate	1 × 10−4
$\boldsymbol{\alpha}$	0.05
$\boldsymbol{\beta}$	0.06
$\boldsymbol{\omega}$	20

Supplementary Table 2. Ablation experiment with density function weight parameter setting

α	β	AP	AP@0.5	AP@0.75
0.03	0.05	0.8605	0.8701	0.8598
0.04	0.05	0.8733	0.8830	0.8725
0.05	0.05	0.8745	0.8842	0.8737
0.06	0.05	0.8769	0.8853	0.8749
**0.05**	**0.06**	**0.8781**	**0.8879**	**0.8773**
0.05	0.07	0.8746	0.8844	0.8738
0.05	0.08	0.8699	0.8796	0.8692
0.05	0.04	0.8699	0.8797	0.8691

Supplementary Table 3. Ablation experiment with loss function weight parameter setting

$\omega_1 : \omega_2$	**Accuracy**	**Precision**	**Recall**	**F1**	**AJI**	**IIOU**
1:3	0.9763	0.8247	0.7513	0.7858	0.5643	0.5561
1:2	0.9811	0.8971	0.7606	0.8227	0.6131	0.6246
1:1	0.9814	0.8884	0.7765	0.8282	**0.6439**	0.6349
1:0	0.9805	0.8804	0.7672	0.8193	0.6299	0.6182
2:1	0.9813	0.8723	0.7926	0.8300	0.6397	0.6300
3:1	0.9816	**0.9043**	0.7634	0.8274	0.6365	0.6321
4:1	0.9815	0.8958	0.7708	0.8280	0.6421	0.6327
5:1	0.9816	0.8816	0.7873	0.8313	0.6390	0.6339
6:1	0.9755	0.8869	0.7857	0.8319	0.6122	0.6254
7:1	**0.9817**	0.8784	0.7927	**0.8329**	0.6326	**0.6383**
8:1	0.9811	0.8690	**0.7930**	0.8287	0.6178	0.6198
9:1	0.9816	0.8810	0.7887	0.8317	0.6293	0.6294

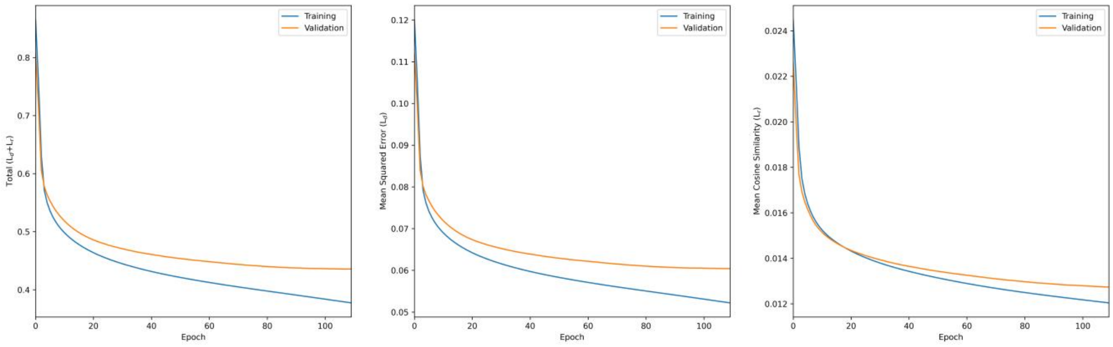

Supplementary Figure 2: The learning curve of the model.

Supplementary Table 4. Segmentation performance of different comparison methods on dataset Platynereis-Nuclei-CBG

Method	Accuracy	Precision	Recall	F1	AJI	IIOU
Cellpose3D	0.94727	0.4830	0.4616	0.4721	0.1552	0.2567
3DCellSeg	0.9797	0.8303	0.7710	0.7996	0.5739	0.5994
EmbedSeg	**0.9821**	0.8069	**0.8232**	0.8150	0.6335	0.6595
ASR	0.9820	**0.8194**	0.8130	**0.8162**	**0.6508**	**0.6711**

Supplementary Table 5. Segmentation performance of different comparison methods on dataset BABB-cleared rat kidney

Method	Precision	Recall	F1	mAP
Cellpose-synth	0.6940	0.7269	0.7098	0.5362
StarDist3D-synth	0.7473	0.4812	0.5840	0.3952
nnU-Net-synth	0.5328	0.2797	0.3650	0.1716
NISNet3D-synth	0.6335	**0.8187**	0.7143	0.5447
ASR	**0.7310**	0.8071	**0.7667**	**0.5671**

References

Girard, L. R., Fiedler, T. J., Harris, T. W., Carvalho, F., Antoshechkin, I., Han, M., ... & Chalfie, M. (2007). WormBook: the online review of Caenorhabditis elegans biology. Nucleic acids research, 35(suppl_1), D472-D475.

Liu, X., Long, F., Peng, H., Aerni, S. J., Jiang, M., Sanchez-Blanco, A., ... & Kim, S. K. (2009). Analysis of cell fate from single-cell gene expression profiles in C. elegans. Cell, 139(3), 623-633.

Chen, A., Wu, L., Winfree, S., Dunn, K. W., Salama, P., & Delp, E. J. (2022). 3d ground truth annotations of nuclei in 3d microscopy volumes. bioRxiv, 2022-09.

Lalit, M., Tomancak, P., & Jug, F. (2021, August). Embedding-based instance segmentation in microscopy. In Medical Imaging with Deep Learning (pp. 399-415). PMLR.
